# Selective TACE with irinotecan-loaded 40 μm microspheres and FOLFIRI for colorectal liver metastases: phase I dose escalation pharmacokinetic study

**DOI:** 10.1186/s12885-019-5862-3

**Published:** 2019-08-01

**Authors:** Toshihiro Tanaka, Takeshi Sato, Hideyuki Nishiofuku, Tetsuya Masada, Shota Tatsumoto, Nagaaki Marugami, Toshio Otsuji, Masatoshi Kanno, Fumikazu Koyama, Masayuki Sho, Kimihiko Kichikawa

**Affiliations:** 1 Department of Radiology, IVR Center, Nara Medical University, 840 Shijo-cho, Kashihara, 634-8522 Japan; 2 Department of Internal Medicine, Dongo Hospital, Yamatotakada, Nara, Japan; 3 Oncology Center, Nara Medical University, Kashihara, Japan; 4 Department of Surgery, Nara Medical University, Nara, Japan

**Keywords:** Liver, Neoplasm metastasis, Hepatic artery, Microspheres

## Abstract

**Background:**

Efficacy of treatments for colorectal liver metastases after failure of first-line chemotherapy is limited. The aim of this study was to prospectively evaluate the feasibility, tolerability, and pharmacokinetics of selective transarterial chemoembolization (TACE) with irinotecan-loaded 40 μm microspheres combined with systemic FOLFIRI for colorectal liver metastases refractory to oxaliplatin regimen.

**Methods:**

The dose escalation study was conducted in three patient groups with different amounts of irinotecan loaded (50, 75 and 100 mg per mL-microspheres). Selective catheterization was performed to embolize subsegments or segments of located tumors using TACE navigation system. FOLFIRI was administrated 7 days after TACE. Plasma concentration was measured before and time points after administration.

**Results:**

Nine patients successfully underwent a total of 22 TACE procedures. Dose-limiting toxicity did not appear at any level. The overall response rate was 55.6%. The median progression free and overall survival were 8.1 and 18.2 months, respectively. The AUC and C_max_ of plasma SN-38 per 1 mg injected irinotecan dose were significantly higher in irinotecan-loaded microspheres compared with FOLFIRI (*P* = 0.009 and *P* <  0.001, respectively).

**Conclusion:**

Selective TACE using 40 μm irinotecan-loaded microspheres combined with systemic FOLFIRI was feasible and safe even when a high dose of irinotecan was loaded. Irinotecan-loaded microspheres resulted in a higher plasma concentration and AUC of SN-38 than treatment with FOLFIRI. Further large scale trials to evaluate the efficacy are mandatory.

**Trial registration:**

University Hospital Medical Information Network (UMIN) Clinical Trials Registry, Registration number; UMIN000015367; Registered date; 08,10,2014.

## Background

Oxaliplatin regimens such as FOLFOX (folinic acid, 5-fluorouracil, and oxaliplatin) combined with biological agents are widely used as first-line treatment for unresectable colorectal liver metastases (mCRC), resulting in high response rates of around 45 to 62% and long progression free survival (PFS) of 10 to 11 months [1, 2]. However, second-line treatment with FOLFIRI (folinic acid, 5-fluorouracil, and irinotecan) or irinotecan combined with bevacizumab is less effective after failure of first-line treatment, with response rates of less than 10% and PFS of only 4 to 5.7 months [1, 3]. Several previous reports showed that transarterial chemoembolization (TACE) with irinotecan-loaded microspheres was effective for treating mCRC after failure of standard systemic chemotherapies [4–6]. However, liver-directed therapy alone could not suppress extrahepatic metastases. Therefore, the combination of TACE and systemic FOLFIRI could be a promising strategy as a second-line therapy.

To date, DC Bead (100 to 300 μm diameter; BTG, London, UK) or DC Bead M1 (75 to 100 μm) have frequently been used as irinotecan-eluting microspheres. However, a previously published literature showed smaller microspheres tend to penetrate further into the target lesion and provide greater drug delivery [7]. Embozene TANDEM (Boston Scientific, Marlborough, MA) is a second generation drug-eluting microsphere available in a smaller, precisely calibrated 40 μm size (http://www.bostonscientific.com/en-EU/products/embolization/embozene_tandem_microspheres.html).

Based on the above background, we performed a clinical study to evaluate the feasibility and efficacy of TACE with 40 μm irinotecan-loaded Embozene TANDEM (TANDEM-IRI) combined with systemic FOLFIRI for mCRC after oxaliplatin regimen failure.

## Methods

This study was a single-center, single-arm, open-label trial (University Hospital Medical Information Network Clinical Trials Registry, registration number UMIN000015367. The study protocol was approved by Nara Medical University Hospital Institutional Review Board (No.14–013). The written informed consents were obtained from all patients in this study. Patients were enrolled from Aug 2015 to May 2017. The doses of irinotecan loaded on the microspheres were escalated in three phases to evaluate tolerability. A navigation system was used to selectively catheterize and embolize the targeted segments or lobes to avoid the risk of proximal biliary complications due to the minute microsphere size. The primary endpoint of this study was to evaluate the safety and recommended dosage of irinotecan in TACE. Tumor response, PFS and overall survival (OS) associated with this combined treatment were also evaluated. The pharmacokinetics of irinotecan and its active metabolite, SN-38, were examined and compared between TANDEM-IRI and systemic FOLFIRI.

### Eligibility criteria

The eligibility criteria for inclusion in this study were as follows: unresectable liver metastases from colorectal adenocarcinoma confirmed histologically; predominant disease with liver metastases (> 50% of total metastatic burden) with or without extrahepatic metastases; failure of first-line chemotherapy (oxaliplatin containing regimen); no lingering side effects from previous therapy and at least a 2-week interval following cessation of the previous therapy. Additional requirements were, Eastern Cooperative Oncology Group performance status (ECOG PS) of 0 to 2 and maintenance of adequate bone marrow, kidney, and cardiac function, meeting the following clinical laboratory tests criteria: white blood cell count ≧3000 /mm^3^ and ≦12,000 /mm^3^, platelet count ≧7.5 × 10^4^ /mm^3^, serum total bilirubin ≦2.5 mg/dl, serum creatinine ≦1.5 mg/dl, BUN ≦25 mg/dl, Prothrombin time ≧50%. Patients were required to be aged ≧20 and ≦80 years, have life expectancy of more than 8 weeks, and provide written informed consent. The exclusion criteria were as follows: previous pancreato-biliary surgery or post endoscopic papillotomy; presence of remarkable AP shunts or AV shunts; UGT1A1 genotypes of the homozygotes and double heterozygotes of *6 and *28 (*6/*6, *28/*28 and *6/*28) to avoid severe complications of irinotecan [8].

#### Dose escalation schedule

According to a 3-by-3 cohort study design, 3 different concentrations of irinotecan were loaded on the microspheres: 50 mg per milliliter microspheres (Level 1), 75 mg per milliliter (Level 2) and 100 mg per milliliter (Level 3). Three patients were enrolled in each level. Starting from Level 1, if there were no dose limiting toxicities noted in all 3 patients, the dose of irinotecan was escalated to the next level in the following enrolled patients. Dose limiting toxicities were shown in Table 1.

**Table 1:** Definition of dose-limiting toxicity

Definition of dose-limiting toxicity	
• Grade 4 neutropenia or thrombocytopenia	
• Grade 3 or 4 neutropenia plus fever higher than 38.0 °C	
• Grade 4 aspartate transaminase and/or alanine transaminase elevation were held over 1 week	
• Serum total bilirubin was ≧5 mg/dl or ≧3 mg/dl was held over 2 weeks	
• Liver abscess, cholangitis and cholecystitis required drainage and surgical resection	
• Non-hematologic toxicities of grade 3 or more (excluding that from disease progression, fatigue, fever, nausea, vomiting, pain and alopecia)	

#### Irinotecan eluting microspheres preparation

Irinotecan solution (Nipponkayaku, Tokyo, Japan) at a dose of 100 mg, 150 mg or 200 mg was mixed with a 2 mL syringe of 40 μm Embozene TANDEM according to the manufacturer’s instructions. The syringe was inverted every 5 min for 30 min. After the irinotecan loading was completed, the supernatant was ejected and the irinotecan-loaded microspheres were mixed with 10 ml of contrast material (Iopamidol 300 mg I/mL).

#### Selective TACE procedure

**Fig. 1:**
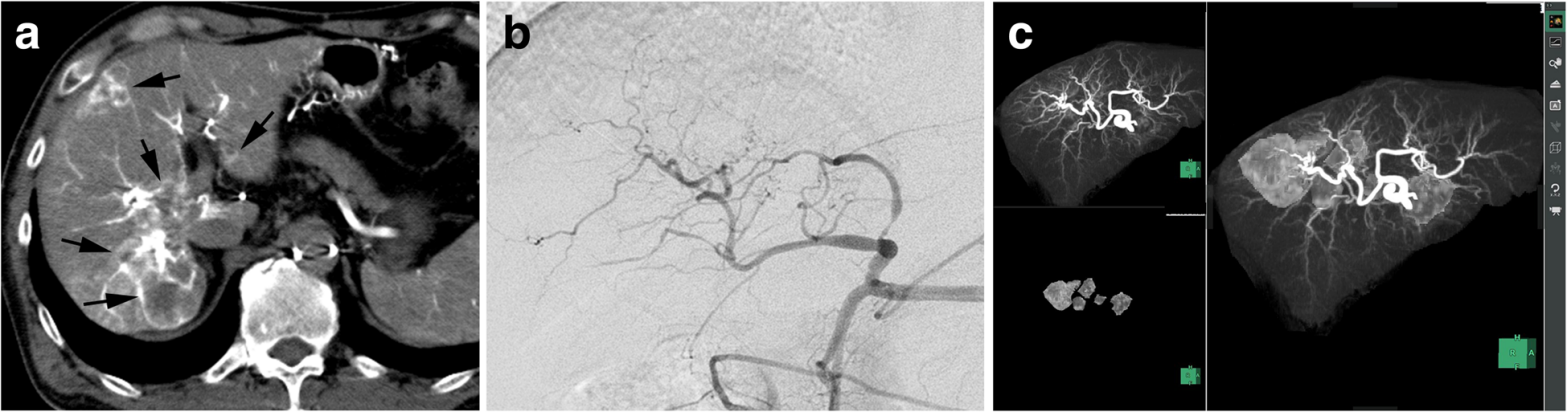
TACE navigation system by using a CT workstation. **a**. CT during contrast injection via the hepatic artery was obtained. Multiple metastases were seen in the right hepatic lobes (arrows). **b**. Hepatic angiography could not depict the tumors. **c**. maximum intensity projection (MIP) image of the hepatic artery and extraction of the tumors were individually created (left side). Fusion of these two images shows the tumor feeding branches of the tumors

A unified CT and angiography system (Angio-CT System, Infinix Activ; Canon Medical Systems) was used. For creation of a TACE navigation image, CT during arterial injection of contrast material via the hepatic artery (CTHA) was obtained and a CT-maximum intensity projection (MIP) image of the hepatic artery was created. The tumors were extracted semi-automatically from the original CTHA image by drawing a line in the maximum cross section of the tumor and fused on the MIP image of the hepatic artery. Image reconstructions were performed with a 3-D CT workstation (SYNAPSE VINCENT; Fujifilm, Tokyo, Japan) (Fig. 1). Using this navigation image, a microcatheter was inserted into the tumor-feeding arteries in the subsegmental levels. If multiple tumors were located in the same segment, segmental TACE was performed. Before performing TACE, CTHA via a microcatheter was obtained to evaluate whether the enhancement areas covered the whole targeted tumors. TANDEM-IRI diluted thirty-fold with contrast material was injected through the microcatheter by hand at a speed of around 1 mL/min. The injection time from starting the injection into the first branch to the end of the TACE procedure was measured. The endpoint of injection was near-stasis or the maximum dose of 2 mL of microspheres. Technical success of the selective TACE procedure was defined as the achievement of microsphere injection covering the tumors, as identified by selective CTHA before TACE.

#### Treatment schedule

**Fig. 2:**
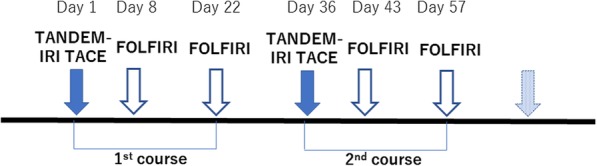
Treatment schedule. TACE with 40 μm irinotecan-loaded microspheres (TANDEM-IRI) was conducted on day 1 followed by FOLFIRI on days 8 and 22. This treatment combination was repeated in 2 or 3 cycles with a 14-day interval

TACE was conducted on day 1 followed by systemic FOLFIRI on day 8. FOLFIRI consisted of a 90-min irinotecan infusion (150 mg/m^2^), according to the results of a phase II study in Japanese patients [3, 9], L-folinic acid (200 mg/m^2^) and 5-fluorouracil (5-FU) bolus (400 mg/m^2^) and a 46-h continuous infusion 5-FU (2400 mg/m^2^). The use of bevacizumab was determined based on potential contraindications (e.g., intact primary tumor with a history of bleeding, recent surgery, and cardiovascular issues). The second FOLFIRI ± bevacizumab was conducted on day 22. TACE followed by FOLFIRI was performed for at least 2 treatment courses (Fig. 2). Repeated TACE procedures were determined for each patient by the treating physician after evaluation of imaging after each course and based on the degree of response and quality of life; i.e. in cases in which the tumors decreased enough to avoid liver disease-related death or there was the possibility to decrease the QOL due to repeated hospitalization, the physicians determined to stop TACE. After termination of TACE therapy, FOLFIRI ± bevacizumab alone was continued every 2 weeks until tumor progression according to RECIST 1.1.

### Clinical evaluation

Toxicities were assessed according to the Common Terminology Criteria for Adverse Event, version 4.0. Contrast enhanced CT was performed after completion of every treatment course to evaluate tumor response according to RECIST 1.1. The targeted lesions included not only liver but also other metastatic sites. Serum carcinoembryonic antigen (CEA) level was also measured after every treatment course. Physical examinations and blood tests were performed before and after every TACE and FOLFIRI. Physical examinations and blood tests were performed before and at 1, 3 and 7 days after every TACE and at 7 days after FOLFIRI. The follow-up period was defined as at least 5 months after initiation of the last treatment course. Duration of survival was calculated from the day of the first TACE.

#### Pharmacological evaluation

To measure serum irinotecan and SN-38 concentrations, 1.5 mL blood was sampled prior to, immediately after, 1 h, 2 h, 3 h, and 18 h after TACE and intravenous infusion of irinotecan in FOLFIRI. Each sample was centrifuged (4 °C 3000 rpm for 10 min) and the plasma was immediately frozen using liquid nitrogen and stored at − 80 °C. The measurements were conducted using liquid chromatography mass spectrometry (LC-MS/MS).

### Statistical analyses

PFS and OS were estimated with Kaplan-Meier methods, and 95% confidence intervals (CI) were provided for proportions using SPSS software (SPSS version 22.2; SPSS, Inc., Chicago, IL, USA). The area under the concentration-time curve (AUC), maximum concentration (C_max_), and time to achieve maximum concentration (t_max_) calculations of the plasma concentration of irinotecan and SN-38 were performed using Pharsight Phoenix WinNonlin 8.0 Modeling Software (Certara G.K.; Princeton, NJ, USA). Statistical significance was defined as a *P* < .05.

## Results

Nine patients were enrolled. The characteristics of the patients are listed in Table 2. Liver metastases were bilobar in all patients. Five patients had extrahepatic metastases: 2 in the lymph node alone, 1 in the lung alone, 1 in the lymph node and lung, and 1 in the lymph node and bone.

**Table 2:** Patient Characteristics (*N* = 9)

Characteristics	N
Gender	
Female	3
Male	6
Mean age (range)	67.3 (55–80)
ECOG performance status	
0	9
Mean longest tumor diameter (mm; range)	48.0 (15–78)
Number of liver metastases, Median (range)	11 (5–15)
n≦5	1
5 < n≦10	3
10≦n	5
Presence of extrahepatic metastases	
no	4
yes	5
Mean serum CEA level	342.4 (range, 6.3–1505.5)

*ECOG* Eastern Cooperative Oncology Group

A total of 22 TACE sessions were performed and all were successful. Selective catheterizations into the subsegment levels were performed in 7 patients (40 subsegmental branches in 18 TACE sessions), the remaining 2 patients received segmental TACE alone in 4 TACE sessions due to multiple tumors located in the same segments. Dose limiting toxicities did not appear at any irinotecan loaded level. The injected doses of microspheres and irinotecan, and the injection times are shown in Table 3. Bevacizumab was combined in 7 patients. The first FOLFIRI started 9.3 ± 1.6 days after TACE and the second FOLFIRI delayed for 1 week in 6 patients. All toxicities during this protocol treatment are listed in Table 4.

**Table 3:** Doses and injection times of TANDEM-IRI and FOLFIRI

	Level 1			Level 2			Level 3		
Patient	1	2	3	4	5	6	7	8	9
Microsphere Dose (ml)	1.0	1.7	1.0	1.0	1.0	1.67	1.5	1.5	2.0
Irinotecan Dose (mg)	50	85	50	75	75	125	150	150	200
Injection Time (hr)	1.05	1.4	0.83	0.95	1.07	1.13	1.45	1.53	1.27
FOLFIRI Dose (mg)	228	246	262	265	214	232.5	234	226	255
Injection Time (hr)	1.5	1.5	1.5	1.5	1.5	1.5	1.5	1.5	1.5

**Table 4:** Adverse events

	Level 1		Level 2		Level 3			
	All	G3	All	G3	All	G3	All (%)	G3 (%)
Hematologic toxicities								
Neutropenia	3	3	2	1	1	1	6 (66.7)	5 (55.6)
Anemia	2	0	1	0	1	1	4(44.4)	1 (11.1)
Thrombocytopenia	0	0	1	0	2	0	3 (33.3)	0 (0.0)
Non-hematologic toxicities								
Elevated AST	3	0	3	1	3	3	9 (100)	3(33.3)
Elevated ALT	1	0	1	1	3	0	5(55.6)	1(11.1)
Increased serum bilirubin	3	0	0	0	0	0	3 (33.3)	0 (0.0)
Fever	0	0	2	0	0	0	2 (22.2)	0 (0.0)
Abdominal Pain	2	0	2	0	3	0	7(77.8)	0 (0.0)

*G3* grade 3

No grade 4 toxicities were found

### Efficacy

**Fig. 3:**
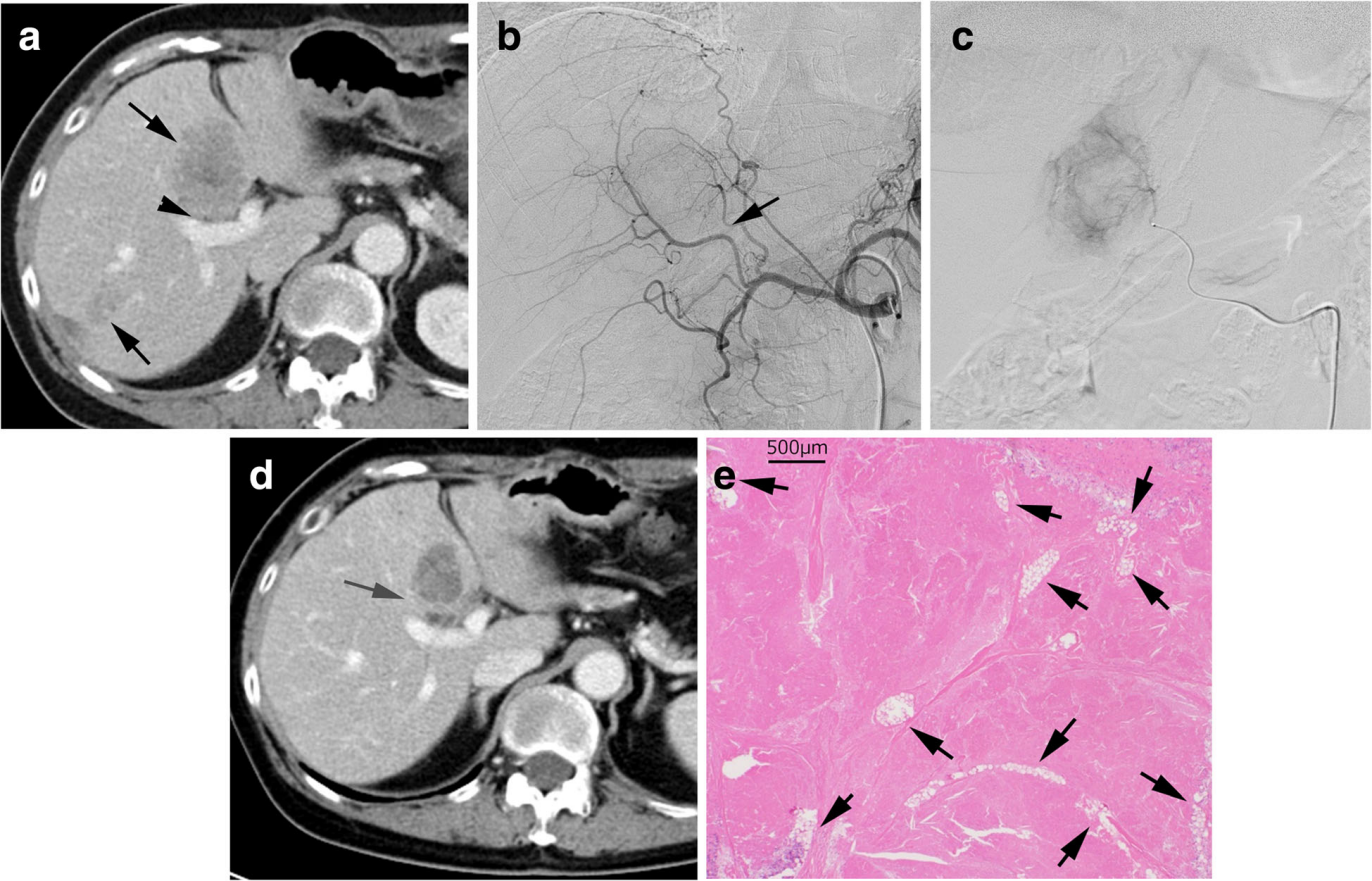
A case received curative surgical resection. This patient had 5 liver metastases, 4 in the left lobe and 1 in the right lobe. There was no extrahepatic metastasis. **a**. CT before TACE shows multiple liver metastases (arrows). A tumor in segment 4 was close to the portal vein (arrowhead). **b**. Hepatic arteriography; the tumor feeding artery was detected (arrow) by the angiography and TACE navigation system. **c**. A catheter was selectively inserted into the tumor feeding artery and 40 μm irinotecan loaded TANDEM was injected. **d**. CT after 3 cycles of the treatment of TACE and FOLFIRI showed the tumor shrunk and was separated from the portal vein (arrow). A bile duct adjacent to the portal vein was slightly dilated. **e**. Histological findings (Hematoxylin and eosin stain) of the resected tumor; almost the whole area of the tumor was necrotic and numerous 40 μm microspheres were seen inside the tumor (arrows)

Twenty-two cycles of combination treatments were performed in 9 patients (mean 2.4, range, 1–3). The overall response rate (complete response + partial response (PR)) was 55.6% (complete response 0, partial response 5, stable disease 4 and progressive disease 0). All PR were achieved due to shrinkage of liver tumors. Shrinkage of extrahepatic metastases was not found. The disease progressions were found in liver tumors alone 2, extrahepatic metastases alone 1 and both of them 6. The numbers of patients who achieved PR in each Level were 2 in Level 1, 1 in Level 2 and 2 in Level 3. The mean time to response was 72 ± 29.9 days. The mean serum CEA levels before and after treatment were 342.4 ng/mL (range, 6.3–1505.5 ng/mL) and 81.4 ng/ml (range, 0.2–563.8 ng/mL), respectively. There was a significant difference between them (*P* = .044). The median follow-up period was 16.9 months (range 4.4–36.9 months). All 9 patients achieved a decrease of the CEA levels by the combined treatment. After the tumor reduction, curative surgical resection was performed in one patient (Fig. 3). All patients had tumor progression. Eight of 9 patients died and the remaining patient has currently been receiving the best supportive care. Median PFS and OS were 8.1 months (95% CI, 6.2–10.1 months) and 18.2 months (95% CI, 11.5–25.3 months), respectively.

### Pharmacokinetics

**Fig. 4:**
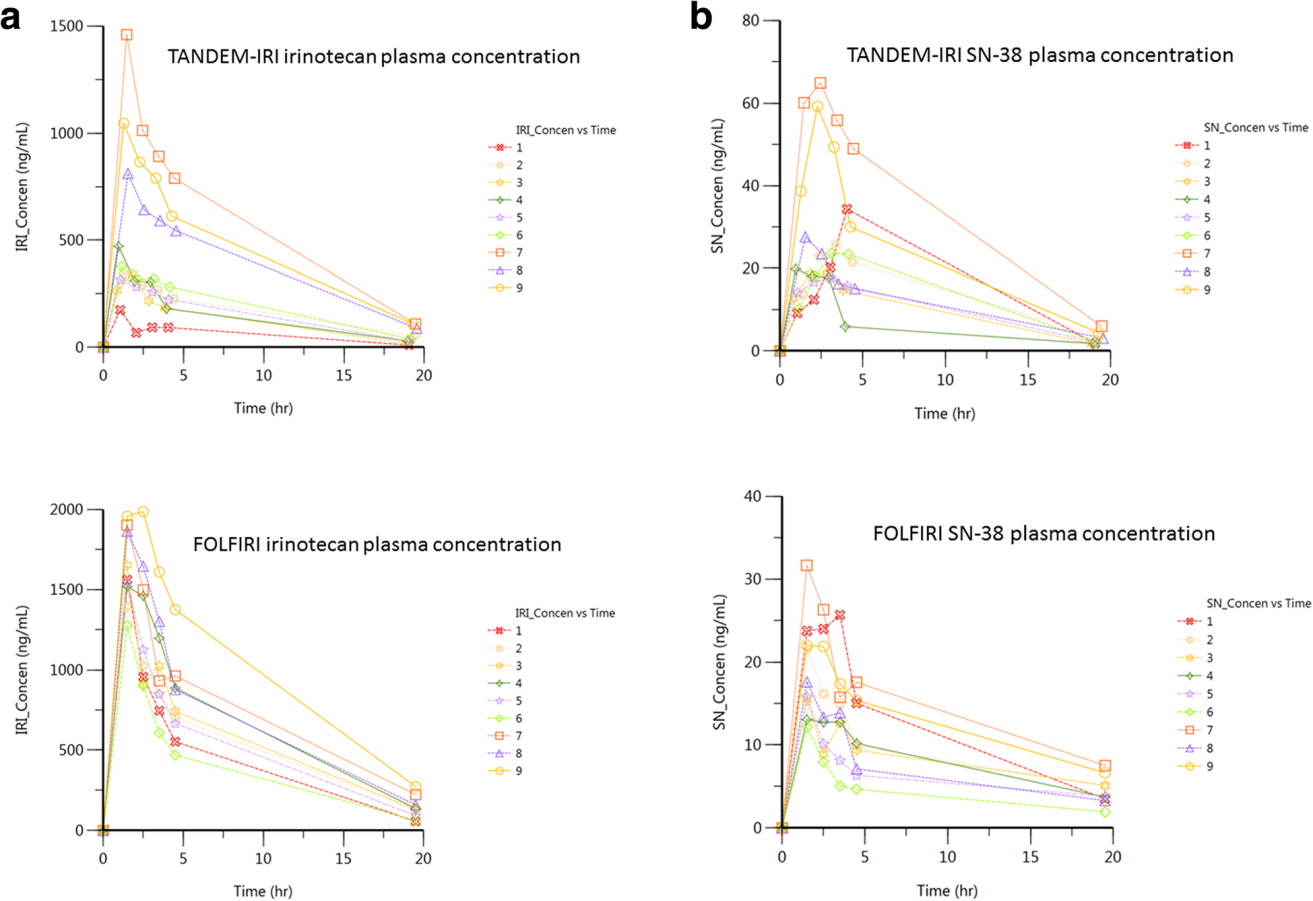
**a**. Irinotecan concentrations in plasma for 18 h after TANDEM-IRI and FOLFIRI. **b**. SN-38 concentrations in plasma for 18 h after TANDEM-IRI and FOLFIRI

Because the dose of TANDEM-IRI received by each patient was dependent on the microsphere volume required to reach near-stasis in addition to the dosage loaded on the microspheres, each patient was treated as an individual data set. The irinotecan and SN-38 concentrations over time are shown for both TANDEM-IRI and FOLFIRI delivery routes (Fig. 4). The concentration of irinotecan peaked after injection, regardless of delivery method, before being metabolized or cleared from the plasma. However, differences of the concentrations of irinotecan at 1 h and 2 h after TANDEM-IRI were milder than those of FOLFIRI. In some cases, the levels at 2 h were higher than 1 h. The concentration of SN-38 tended to increase over the first 2 h in TANDEM-IRI or plateau over the first 2 h in the case of FOLFIRI.

The mean AUC and C_max_ of irinotecan and SN-38, and those values calculated as per 1 mg injected irinotecan dose (AUC/Dose and C_max_/Dose) were shown in Table 5. The AUC/Dose and C_max_/Dose of plasma SN-38 were significantly higher in TANDEM-IRI compared with FOLFIRI (*P* = 0.009 and *P* <  0.001, respectively), while there were no significant differences in those of plasma irinotecan. The mean t_max_ of SN-38 delivered by TANDEM-IRI and FOLFIRI were 2.95 ± 1.35 h and 1.5 ± 0.0 h, respectively.

**Table 5:** Comparison of the pharmacokinetics of irinotecan and SN-38

	TANDEM-IRI	FOLFIRI	*P* value
AUC of irinotecan (hr*ng/ml)	4327.1 ± 2919.1	8958.1 ± 4484.1	N.S.
AUC/Dose of irinotecan	37.8 ± 12.8	36.9 ± 17.0	N.S
AUC of SN-38 (hr*ng/ml)	269.9 ± 148.3	165.7 ± 90.3	N.S
AUC/Dose of SN-38	3.01 ± 1.87	0.68 ± 0.36	0.009
C_max_ of irinotecan (hr*ng/ml)	624.7 ± 469.7	1626.0 ± 264.1	< 0.001
C_max_/Dose of irinotecan	5.4 ± 2.4	6.8 ± 1.2	N.S
C_max_ of SN-38 (hr*ng/ml)	30.4 ± 17.1	18.8 ± 6.3	N.S
C_max_/Dose of SN-38	0.32 ± 0.11	0.08 ± 0.03	< 0.001

**Fig. 5:**
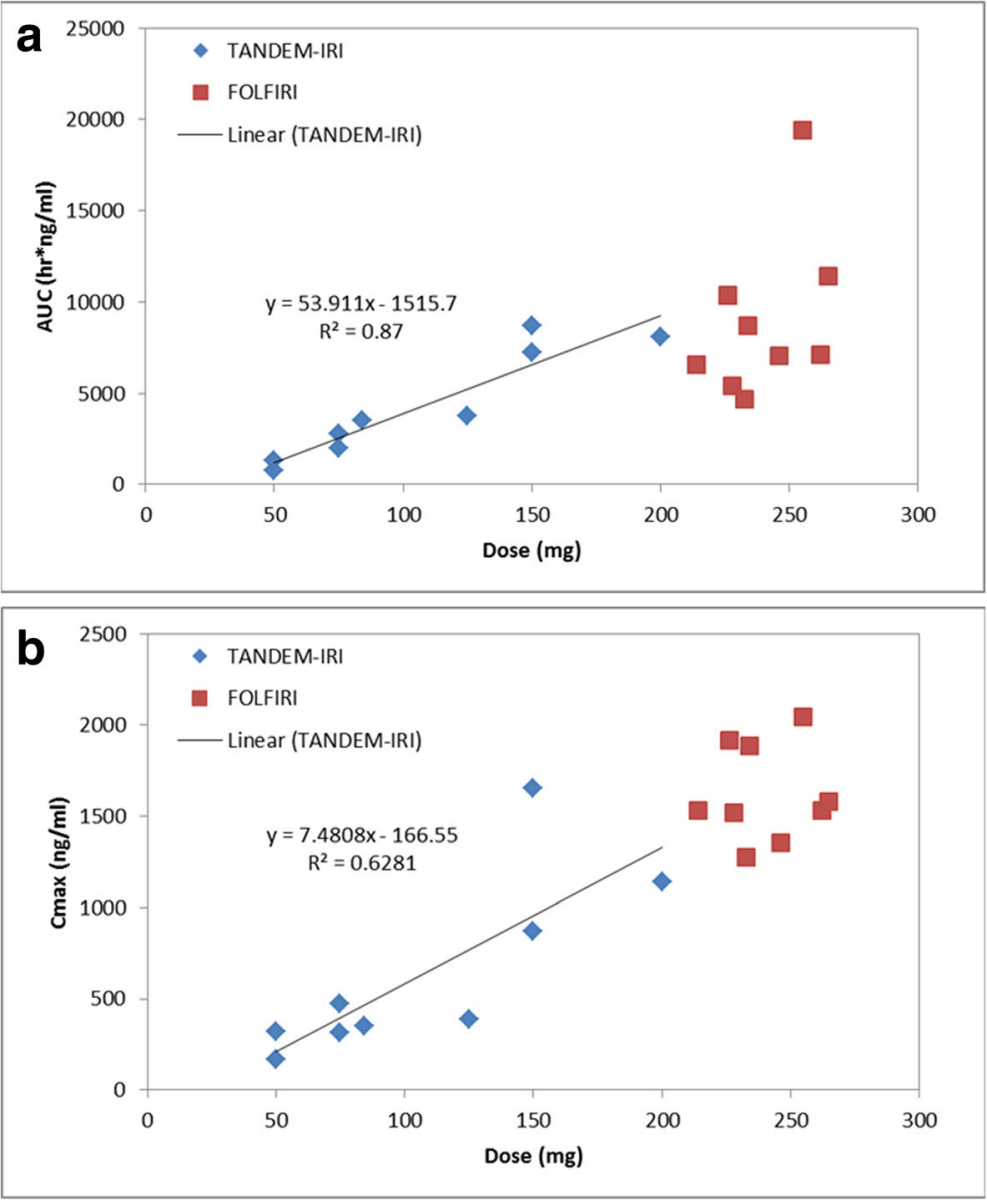
**a**. Correlation between injected irinotecan doses and AUC values after TANDEM-IRI (blue) and FOLFIRI (red). There was a linear correlation after TANDEM-IRI. **b**. Correlation between irinotecan injected doses and C_max_ values after TANDEM-IRI (blue) and FOLFIRI (red). There was a linear correlation after TANDEM-IRI

Each patient received variable dosages due to achieved stasis levels. The C_max_ and total drug exposure, quantified as AUC, are typically dependent on dosage delivered. To determine the irinotecan dose dependency relationship, AUC and C_max_ versus Dose plots were generated in Fig. 5. TANDEM-IRI displayed a linear irinotecan dose dependency in both AUC and C_max_. There was not enough dosage variability in the FOLFIRI data set to discern a relationship, however, the data points appear to align with the linear dose dependency established by TANDEM-IRI.

## Discussion

Our prospective study confirmed the safety of irinotecan-loaded microspheres for TACE in addition to FOLFIRI. A previously published animal study showed that there was no irinotecan over-dosing of the systemic circulation 24 h after TACE combined with intravenous irinotecan infusion [10]. In our study, grade 3 adverse events of AST elevation and neutropenia were found. The former could be related to TACE and the latter to FOLFIRI. Both are well known events caused by these individual treatments and no synergistic toxicities were found [11, 12].

Previously published literature showed the advantages of smaller microspheres, 70–150 μm in size, when compared with 100–300 μm. There was a lower percentage of treatments terminating in complete stasis. Also, this report showed an increased objective response rate at 12 months in the small bead group [7]. Another in vivo study demonstrated that 70–150 μm beads resulted in a more distal embolization in liver tissue and provided greater coverage of the tumor periphery [13, 14]. We used microspheres 40 μm in size, which are the smallest currently available for clinical use. Theoretically, 40 μm microspheres could have greater advantages than 70–150 μm beads, which could penetrate further into fine tumor feeding arteries and achieve better drug distribution.

However, 40 μm microspheres could migrate into peribiliary arteries with the risk of causing proximal biliary tract damage [15]. Therefore, we performed selective catheterization to avoid such complications. As a result, in our study, no major TACE-related complications were seen. Further large scale studies are needed to check the safety of small microspheres for bile duct damage. Also, a risk of migration of small microspheres into the lung or the brain should be discussed. Previous report discussed the necessity of ^99m^Tc-*MAA* evaluation before small microsphere injection. This issue remains controversial [16].

Our TACE navigation system with a fused image was useful to detect the tumor-feeding arteries. In all cases, the enhancement area in selective CTHA covered the whole tumor, which determined a 100% technical success rate although CT after TACE was not obtained.

Regarding the irinotecan doses in TANDEM, the manufacturer’s instructions recommend a loading dose of 50 mg irinotecan per 1 mL microspheres (http://www.bostonscientific.com/en-EU/products/embolization/embozene_tandem_microspheres.html). However, to date, there is no data regarding the possibility of further dose loading. Our pharmacological data demonstrated a linear dose dependency in AUC and C_max_, which suggested that TANDEM could load 200 mg irinotecan per 1 mL microspheres. The tolerability of 200 mg-loaded TANDEM-IRI was also shown in this study although 200 mg irinotecan in 2 mL microspheres was fully administrated in only one patient.

Our study compared the pharmacokinetics between irinotecan-loaded microspheres and intravenous infusion in the same patients. Our results showed similar irinotecan release via both delivery methods, although injection times were different. However, the irinotecan concentrations at 1 h and 2 h after TANDEM-IRI showed slower drug release. The AUC and C_max_ of SN-38, the active metabolite of irinotecan, in dose-normalization had significantly higher values in TANDEM-IRI than in FOLFIRI, which could achieve more effective anti-tumor effect. This suggests that irinotecan delivered directly into the liver was converted more effectively to SN-38 compared with the systemic delivery. Previous literature reported that a low dose continuous infusion of irinotecan showed higher value of metabolic SN-38 / irinotecan AUC ratio when compared with a commonly used short infusion schedule [17]. The slow drug release of TANDEM-IRI and lower dose administration could also lead to effective metabolism. The t_max_ of SN-38 in TANDEM-IRI was longer than that of FOLFIRI. This could suggest the slow irinotecan release of TANDEM-IRI.

Previous literature argued that lobar delivery of irinotecan-loaded microspheres could be more effective than selective delivery for the treatment of occult liver metastases and the activation of irinotecan to SN-38 by normal hepatocytes [18]. However, a study using an animal liver tumor model showed that selective TANDEM-IRI achieved a high tumor concentration of SN-38 [19]. Our current study also demonstrated that irinotecan successfully converted SN-38 by the selective injection. The treatment of occult metastases may not be relevant in many patients with advanced diseases.

The combination of TANDEM-IRI TACE with FOLFIRI achieved the response rate of 55.6% and PFS of 8.1 months in patients who had previously failed an oxaliplatin regimen. These results might be due to lower disease burden and patient selection.

There are several limitations in this current study. First, this was a study with a small sample size. Only patients with PS 0 were treated although patients with PS 0–2 were eligible in this study. Therefore, evaluations in this treatment for patients with severe PS are needed. Second, various doses of irinotecan were delivered by TANDEM-IRI because the endpoint of injection was stasis of the hepatic blood flow. Consequently, the maximum dose of 200 mg irinotecan was fully administrated in only one patient. A larger scale phase II study is need.

## Conclusions

This dose escalation study indicated TACE using irinotecan loaded TANDEM at a dose of 100 mg per 1 mL microspheres was tolerable and safe combined with FOLFIRI. Selective TACE for mCRC was technically feasible using a TACE navigation system. TANDEM-IRI resulted in a higher plasma concentration and AUC of SN-38 than treatment with FOLFIRI.

## Data Availability

The datasets used and/or analysed during the current study are available from the corresponding author on reasonable request.

## Abbreviations

AUC: Area under the concentration-time curve
C_max_: maximum concentration
CTHA: CT during arterial injection of contrast material via the hepatic artery
ECOG: Eastern Cooperative Oncology Group
mCRC: colorectal liver metastases
MIP: Maximum intensity projection
OS: Overall survival
PFS: Progression free survival
PS: Performance status
TACE: Transarterial chemoembolization
TANDEM-IRI: Irinotecan-loaded Embozene TANDEM
t_max_: time to achieve maximum concentration

## References

[1] Tournigand C, André T, Achille E (2004). FOLFIRI followed by FOLFOX6 or the reverse sequence in advanced colorectal cancer: a randomized GERCOR study. J Clin Oncol.

[2] Yamazaki K, Nagase M, Tamagawa H (2016). Randomized phase III study of bevacizumab plus FOLFIRI and bevacizumab plus mFOLFOX6 as first-line treatment for patients with metastatic colorectal cancer (WJOG4407G). Ann Oncol.

[3] Kuramochi H, Ando M, Itabashi M (2017). Phase II study of bevacizumab and irinotecan as second-line therapy for patients with metastatic colorectal cancer previously treated with fluoropyrimidines, oxaliplatin, and bevacizumab. Cancer Chemother Pharmacol.

[4] Fiorentini G, Aliberti C, Tilli M (2012). Intra-arterial infusion of irinotecan-loaded drug eluting beads (DEBIRI) versus intravenous therapy (FOLFIRI) for hepatic metastases from colorectal cancer: final results of a phase III study. Anticancer Res.

[5] Bhutiani Neal, Akinwande Olaguoke, Martin Robert C. G. (2015). Efficacy and Toxicity of Hepatic Intra-Arterial Drug-Eluting (Irinotecan) Bead (DEBIRI) Therapy in Irinotecan-Refractory Unresectable Colorectal Liver Metastases. World Journal of Surgery.

[6] Martin RC, Joshi J, Robbins K (2011). Hepatic intra-arterial injection of drug-eluting bead, irinotecan (DEBIRI) in unresectable colorectal liver metastases refractory to systemic chemotherapy: results of multi-institutional study. Ann Surg Oncol.

[7] Akinwande OK, Philips P, Duras P, Pluntke S, Scoggins C, Martin RC (2015). Small versus large-sized drug-eluting beads (DEBIRI) for the treatment of hepatic colorectal metastases: a propensity score matching analysis. Cardiovasc Intervent Radiol.

[8] Dean L. Irinotecan therapy and UGT1A1 genotype. Medical Genetics Summaries [Internet]. Bethesda (MD): National Center for Biotechnology Information (US); 2012-2015 May 27 [updated 2018 Apr 4] 28520360

[9] Kochi M, Akiyama Y, Aoki T (2013). FOLFIRI plus bevacizumab as a first-line treatment for Japanese patients with metastatic colorectal cancer: a JACCRO CC-03 multicenter phase II study. Cancer Chemother Pharmacol.

[10] Lewis AL, Holden RR, Chung ST (2013). Feasibility, safety and pharmacokinetic study of hepatic administration of drug-eluting beads loaded with irinotecan (DEBIRI) followed by intravenous administration of irinotecan in a porcine model. J Mater Sci Mater Med.

[11] Martin RC, Howard J, Tomalty D (2010). Toxicity of irinotecan-eluting beads in the treatment of hepatic malignancies: results of a multi-institutional registry. Cardiovasc Intervent Radiol.

[12] Fuse N, Doi T, Ohtsu A (2008). Safety of irinotecan and infusional fluorouracil/leucovorin (FOLFIRI) in Japan: a retrospective review of 48 patients with metastatic colorectal cancer. Int J Clin Oncol.

[13] Lewis AL, Dreher MR, O'Byrne V (2016). DC bead M1: towards an optimal trans catheter hepatic tumour therapy. J Mater Sci Mater Med.

[14] Dreher MR, Sharma KV, Woods DL (2012). Radiopaque drug-eluting beads for transcatheter embolotherapy: experimental study of drug penetration and coverage in swine. J Vasc Interv Radiol.

[15] Demachi H, Matsui O, Takashima T (1991). Scanning electron microscopy of intrahepatic microvasculature casts following experimental hepatic artery embolization. Cardiovasc Intervent Radiol.

[16] Bonomo G, Pedicini V, Monfardini L (2010). Bland embolization in patients with unresectable hepatocellular carcinoma using precise, tightly size-calibrated, anti-inflammatory microparticles: first clinical experience and one-year follow-up. Cardiovasc Intervent Radiol.

[17] Herben VM, Schellens JH, Swart M (1999). Phase I and pharmacokinetic study of irinotecan administered as a low dose, continuous intravenous infusion over 14 days in patients with malignant solid tumors. J Clin Oncol.

[18] Jones RP, Dunne D, Sutton P (2013). Segmental and lobar administration of drug-eluting beads delivering irinotecan leads to tumourdestruction: a case-control series. HPB (Oxford).

[19] Tanaka T, Nishiofuku H, Hukuoka Y (2014). Pharmacokinetics and antitumor efficacy of chemoembolization using 40 μm irinotecan-loaded microspheres in a rabbit liver tumor model. J Vasc Interv Radiol.

